# SDPR expression in human trabecular meshwork and its potential role in racial disparities of glaucoma

**DOI:** 10.1038/s41598-024-61071-w

**Published:** 2024-05-04

**Authors:** Ying-Bo Shui, Ying Liu, Andrew J. W. Huang, Carla J. Siegfried

**Affiliations:** Department of Ophthalmology and Visual Sciences, Washington University School of Medicine, St. Louis, MO 63110 USA

**Keywords:** Glaucoma, Mechanisms of disease

## Abstract

In order to identify how differential gene expression in the trabecular meshwork (TM) contributes to racial disparities of caveolar protein expression, TM dysfunction and development of primary open angle glaucoma (POAG), RNA sequencing was performed to compare TM tissue obtained from White and Black POAG surgical (trabeculectomy) specimens. Healthy donor TM tissue from White and Black donors was analyzed by PCR, qPCR, immunohistochemistry staining, and Western blot to evaluate SDPR (serum deprivation protein response; Cavin 2) and CAV1/CAV2 (Caveolin 1/Caveolin 2). Standard transmission electron microscopy (TEM) and immunogold labeled studies were performed. RNA sequencing demonstrated reduced *SDPR* expression in TM from Black vs White POAG patients’ surgical specimens, with no significant expression differences in other caveolae-associated genes, confirmed by qPCR analysis. No racial differences in *SDPR* gene expression were noted in healthy donor tissue by PCR analysis, but there was greater expression as compared to specimens from patients with glaucoma. Analysis of SDPR protein expression confirmed specific expression in the TM regions, but not in adjacent tissues. TEM studies of TM specimens from healthy donors did not demonstrate any racial differences in caveolar morphology, but a significant reduction of caveolae with normal morphology and immuno-gold staining of SDPR were noted in glaucomatous TM as compared to TM from healthy donors. Linkage of SDPR expression levels in TM, POAG development, and caveolar ultrastructural morphology may provide the basis for a novel pathway of exploration of the pathologic mechanisms of glaucoma. Differential gene expression of SDPR in TM from Black vs White subjects with glaucoma may further our understanding of the important public health implications of the racial disparities of this blinding disease.

## Introduction

Primary open-angle glaucoma (POAG) is an optic neuropathy that disproportionately affects Black (B) persons with earlier onset, faster progression, and higher prevalence than White (W)^1–4^. Although socioeconomic factors may contribute, the cellular mechanisms underlying this racial difference remain unclear. Genome-wide association studies (GWAS) of W individuals have identified significant associations with *CAV1/CAV2* (Caveolin 1/Caveolin 2) gene polymorphisms and POAG^5,6^. However, gene expression in ocular tissues does not consistently correspond to these variants, particularly in diverse ancestral cohorts^7^. Thus, investigating specific gene expression in the trabecular meshwork (TM) may be crucial to identifying the genetic basis of POAG risk associated with intraocular pressure (IOP) dysregulation and racial disparities^8^. Using RNAseq data from fresh TM specimens of POAG patients, we observed significantly lower SDPR (serum deprivation protein response; Cavin 2) in B compared to W patients. This disparate expression of SDPR may underlie TM dysfunction and enable personalized therapies for POAG.

## Main

Increased intraocular pressure (IOP) is the most significant risk factor for POAG and lowering IOP is the only current means of therapy^9,10^. The TM in the anterior chamber angle serves as the primary site for conventional aqueous humor outflow, regulating IOP by modulating varying tissue-specific resistance^11^. Despite extensive research efforts, the specific cellular mechanisms responsible for elevated IOP in glaucoma remain unclear^11^.

Emerging evidence suggests that caveolae, sub-microscopic organelles of the plasma membrane initially identified in the 1950s, play a key role in IOP regulation within the TM^12,13^. These cellular structures are involved in various cellular functions and diseases, particularly in response to mechanosensitive stress and fluid pressure, leading to remodeling of the extracellular environment^14,15^. Caveolae are abundantly expressed in the cells of the conventional aqueous outflow pathway including Schlemm’s canal (SC) and the TM^16,17^. Several physiological processes are associated with caveolar function including mechanotransduction ^18^ and cellular signaling^19^. Caveolins (CAV1, CAV2) and cavins (Cavin 1, 2, 3, 4) are involved in caveolar biosynthesis and morphology^20,21^. Several studies have implicated CAV1 modulation in murine IOP regulation^15,22^, as demonstrated in global CAV1 knock out mice and rescue with TM expression of CAV1^23^. The family of caveolar proteins play an essential role in IOP regulation in the TM and further research is needed to understand the biomechanical mechanisms involved.

SDPR is a critical regulator of the formation of caveolar structures^21^. Particularly, its expression varies in different ocular tissues, indicating potential tissue-specific caveolar functions that may not be adequately addressed in GWAS^5,6^. We hypothesize that variations in SDPR expression in the TM may contribute to IOP regulation and risk of glaucoma, with potential racial disparities in its impact on caveolar structure and function. Despite these observations, the exact mechanisms of caveolar function in health and disease, particularly in the context of glaucoma, remain largely unexplored^15,24^.

**Figure 1:**
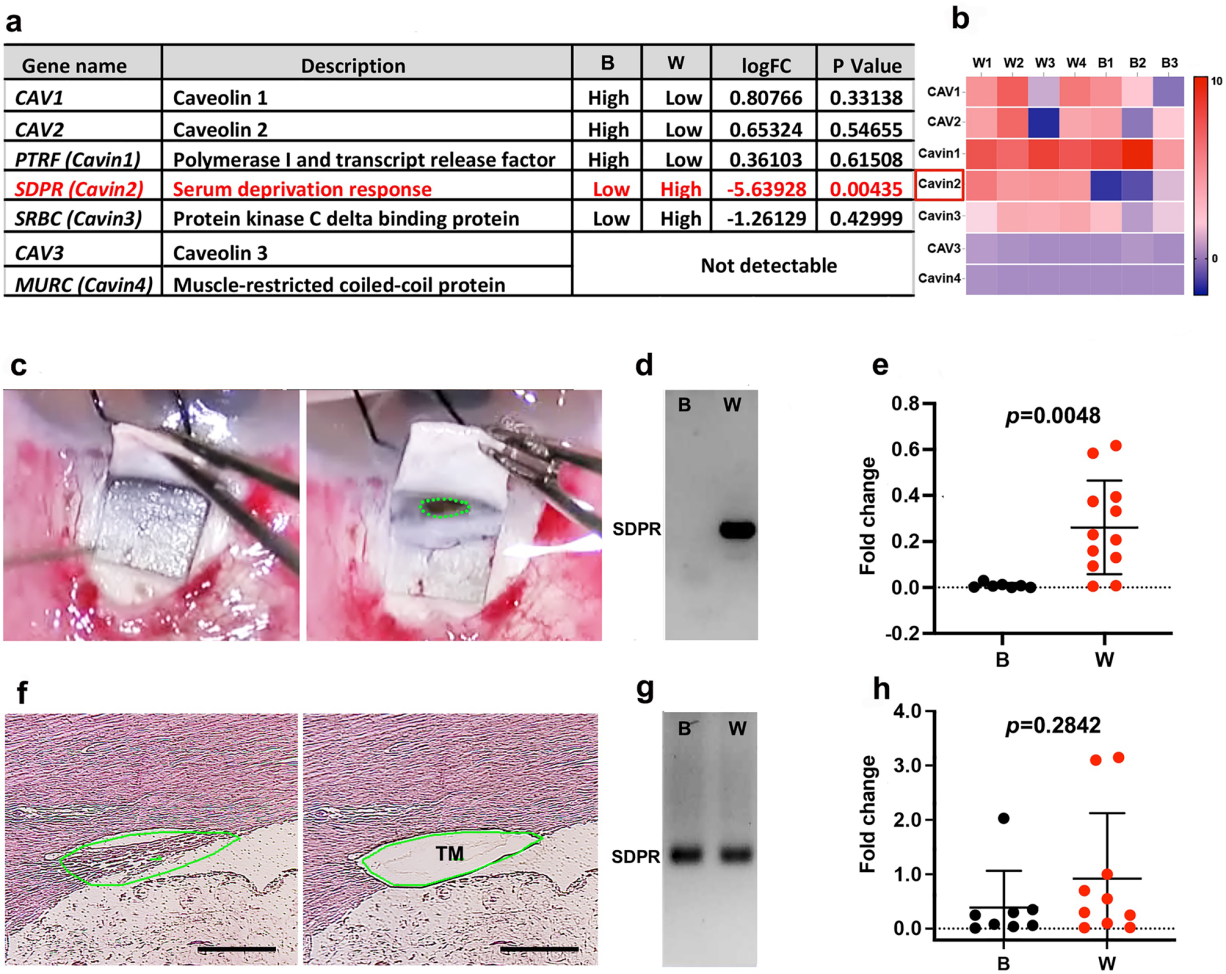
Differential gene expression of trabecular meshwork. (**a**) RNAseq analysis of caveolae-related gene expression from glaucomatous trabecular meshwork (TM) tissues Black (B) = 3; White (W) = 4. (**b**) RNAseq Heat map. (**c**) Intraoperative image of TM specimen obtained during glaucoma surgery (trabeculectomy; green line delineates area of excised tissue). (**d**) Standard PCR of pooled glaucomatous TM (n = 4/lane). (**e**) qPCR data of glaucomatous TM (B = 7, W = 12). (**f**) Laser dissection technique used to precisely isolate TM tissue from a White donor eye by first delineating the TM region (green outlined area), and then excising it (void within green outline). This technique was consistently applied to collect TM samples from both White and Black healthy donors. (**g**) Standard PCR of pooled donor TM (n = 3/lane). (**h**) qPCR of donor TM (B = 8, W = 10). Mean ± SD. LogFC: Log Fold Change. B: Black. W: White Bar: 200 μm).

We conducted RNA sequencing (RNAseq) analysis on specimens obtained from patients undergoing glaucoma surgery (trabeculectomy), stratifying self-reported B and W individuals (Fig. 1, legend). The RNAseq data revealed that among the caveolae-related gene family, only SDPR expression was significantly reduced (5.6 log-fold) in B compared to W patient TM specimens (Fig. 1a,b). In contrast, no significant racial differences were found in the expression of other caveolae-related genes such as CAV1-2, Cavin1 and 3. CAV3 and MURC (Cavin4), both specific to myocytes, were not detected in TM tissue^15,25,26^. To validate RNAseq results, we collected additional tissue specimens containing TM from POAG patients undergoing trabeculectomy surgery (Fig. 1c), pooling them together with stratification by racial background (B and W). Using conventional polymerase chain reaction (PCR) analysis, we confirmed significant reduction of SDPR expression in B patients compared to W patients (Fig. 1d). In contrast, no racial differences were observed for CAV1-2, Cavin1, and Cavin3 genes (data not shown). To support this finding, we performed quantitative PCR (qPCR) analysis (Fig. 1e), which further confirmed significant racial disparities in SDPR expression among individual patients (p = 0.0048). Although conventional PCR and qPCR are different approaches, both PCR methods indicate analogous results of reduced expression of SDPR in B vs W POAG patient specimens. POAG patient demographics are provided in Supplementary Table 1. Of note, there were no significant differences in age, pre-operative glaucoma medication usage, or glaucoma severity^27^ in the B compared to the W group.

In our investigation of non-glaucomatous TM, we utilized TM tissues obtained from donor corneal rims as healthy control. To mitigate potential postmortem effects, we exclusively employed donor tissues preserved within 48 h postmortem and stored in Optisol-GS (Bausch & Lomb, Inc) for less than 6 days. Using laser microdissection technique (Fig. 1f), we meticulously collected entire TM tissue sections and performed PCR with pooled samples, as well as qPCR on individual samples. We did not find any significant differences in SDPR gene expression between B and W healthy donors (Fig. 1g,h; p = 0.284). Comparing SDPR gene expression between POAG specimens and healthy TM is challenging due to differences in the conditions under which the specimens were collected. Specifically, the specimens vary in terms of their preservation methods—fresh versus postmortem changes with Optisol-GS preservation. To account for this variability, we normalized the data using the housekeeping gene GAPDH as a reference. Upon normalization, we observed that TM from individuals with POAG generally exhibited lower SDPR gene expression compared to TM from healthy donors. Notably, a statistically significant reduction in SDPR gene expression was observed exclusively in the POAG patient TM group, and not in the healthy donor TM group (Fig. 1e,h).

**Figure 2:**
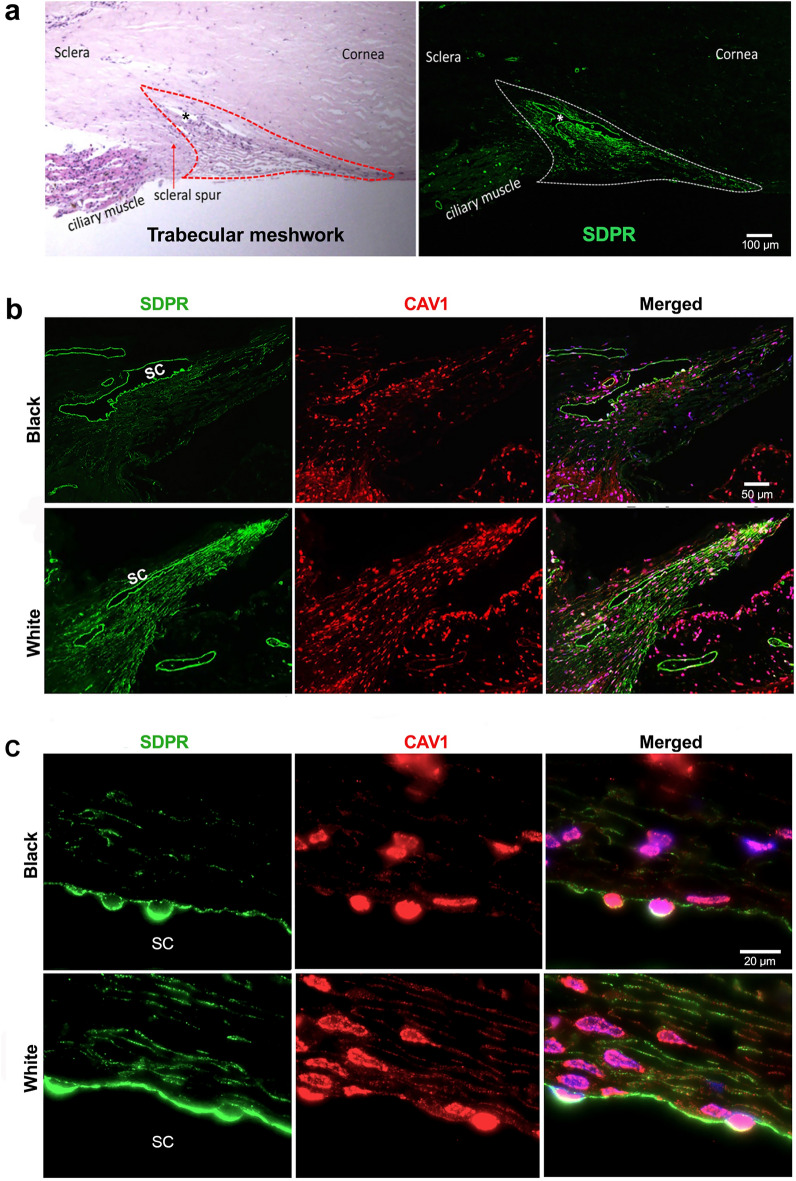
Trabecular meshwork protein expression. (**a**) Left: Photomicrograph of anatomical landmarks of the anterior chamber angle (Hematoxylin–Eosin staining) Right: SDPR immunohistochemistry staining demonstrating strong expression in donor TM and adjacent blood vessels in the anterior chamber angle. Bar: 100 µm (**b**) Immunohistochemistry staining in donor TM and adjacent tissues demonstrating SDPR (green) expressions specifically located in the TM region and CAV1 (red) protein expressions throughout the anterior chamber angle. Upper panels (Black donor), lower panels (White donor). Bar: 50 µm. (**c**) High-magnification images (oil immersion lens) of juxtacanalicular TM with SDPR (green) localized to the cell membrane, while CAV1 (red) expression is observed in the cell membrane, cytoplasm and some nuclear staining. Upper panels (Black donor), lower panels (White donor). Bar: 20 µm.

Utilizing TM samples harvested from healthy corneal donors, we also investigated SDPR protein expressions, which served as a basis for our study of racial disparities (Fig. 2, legend). Immunohistochemical staining using SDPR antibody revealed strong and specific SDPR expression in the TM regions, including juxtacanalicular, corneoscleral, and uveoscleral meshwork (Fig. 2a). However, adjacent structures such as the cornea, sclera, and ciliary body exhibited minimal or no SDPR staining. A noted exception was the blood vessels that were used as a positive control, consistent with the demonstration of high SDPR expression in systemic vasculature^20^. Concurrently, CAV1, which is commonly assessed in caveolar research^28^, did not display TM-specific expression. As shown in Fig. 2b (red), CAV1 expression was also observed in adjacent anatomical structures such as the iris and ciliary body, but not in the corneal stroma. We further performed immunohistochemistry staining to compare SDPR expression in TM between B and W healthy individual donors. We found that in B donor tissue, SDPR expression was significantly lower in the corneoscleral and uveoscleral trabecular meshwork regions compared to W donors. However, SDPR expression in SC and juxtacanalicular trabecular meshwork (JCT) regions remained preserved in both racial groups (Fig. 2b). Additionally, we assessed another caveolar family member, CAV1, in the same samples. It appeared that CAV1 expression was lower in B donors compared to W donors in the TM regions, and minimal expression was observed in SC/JCT (Fig. 2b). Utilizing an oil immersion lens to capture high-magnification images, we were able to discern additional details concerning the expression patterns of SDPR and CAV1 proteins (Fig. 2c). Specifically, SDPR expression was predominantly localized to the cellular membrane. Moreover, expression in the SC/JCT region was more robust than in corneoscleral and uveoscleral TM regions. In contrast, CAV1 expression was more ubiquitous and not limited to the cellular membrane. CAV1 was also present in the cytoplasm and, consistent with previous reports, displayed expression within the nucleus^29^.

**Figure 3:**
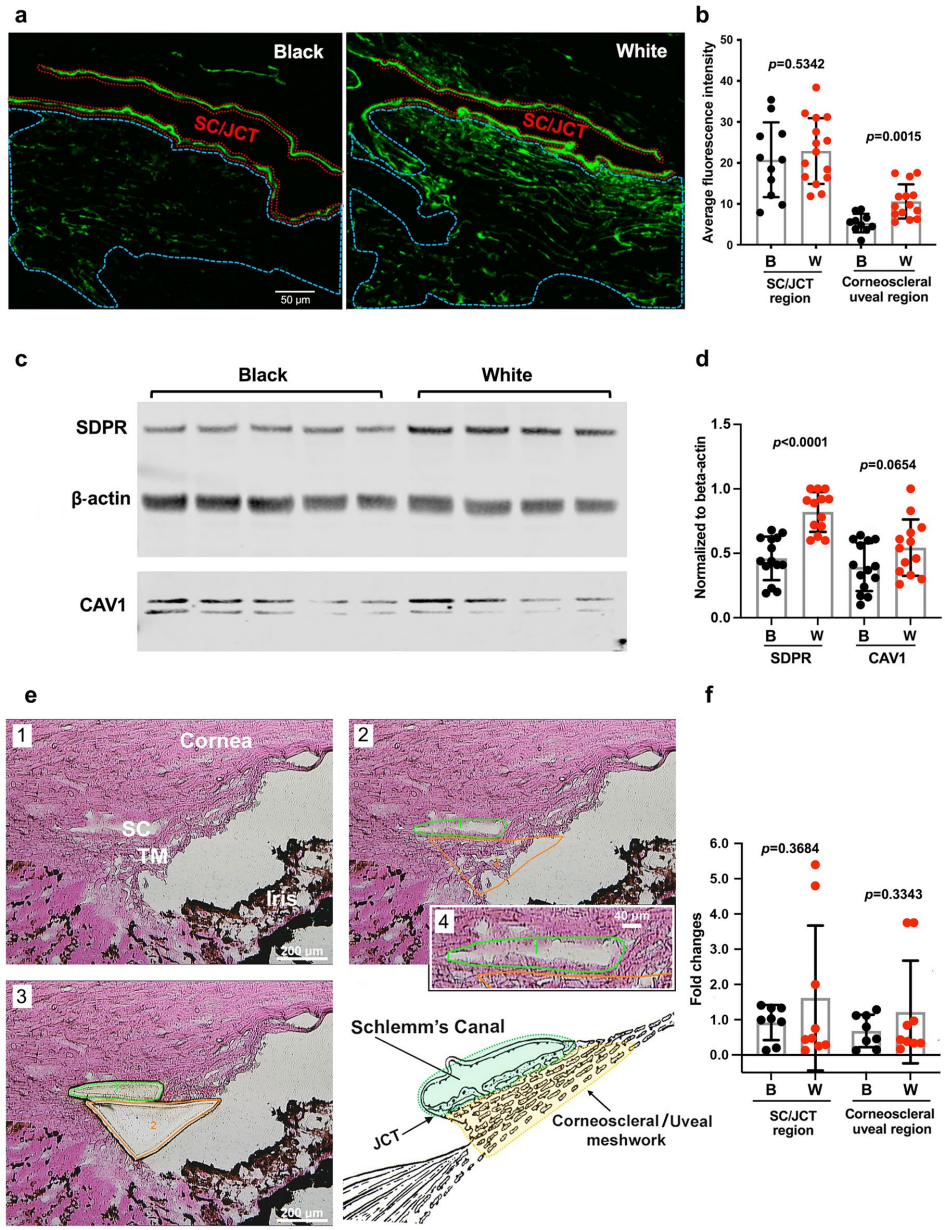
SDPR and CAV1 quantitative protein and qPCR analysis. (**a**) Immunochemistry staining of SDPR and CAV1. Red line delineates Schlemm’s canal (SC) with juxtacanalicular TM (JCT), blue line delineates corneoscleral meshwork regions. Bar: 50 μm (**b**) Quantitative analysis using Image J (Version 1.51a, NIH, USA). Mean SDPR fluorescence intensity (B = 11; W = 15). (**c**) Western blot data. (**d**) Normalized SDPR and CAV1 expressions based on beta-actin expression. B = 14; W = 13. Mean ± SD. (**e**) Examination of SDPR gene expression in specific locations by precise laser microdissection. e1-Eosin Y stain of anterior chamber angle structures and TM region. e2-Delineation of TM for laser microdissection. Schlemm’s canal (SC) and juxtacanalicular TM (JCT; green), corneoscleral and uveoscleral TM (orange). e3-Distinct dissection of SC/JCT (green) and corneoscleral and uveoscleral (orange) regions for RNA analysis. e4-High-magnification image of SC/JCT and diagram illustrating specific TM areas. (**f**) qPCR results of RNA isolations from SC/JCT and corneoscleral and uveoscleral regions. B = 8, W = 10. Bar: 200 μm.

We then performed quantitative analysis of SDPR and CAV1 (Fig. 3, legend) by immunohistochemistry staining using Image J analysis, dividing fluorescence expression areas into SC/JCT and corneoscleral/uveoscleral TM regions (Fig. 3a,b). SDPR protein staining was significantly reduced in the corneoscleral and uveoscleral trabecular meshwork regions of B healthy donors compared to W donors (Fig. 3b; p = 0.0015), while no statistical difference was observed in SC/JCT (p = 0.5342). No significant racial difference was found in CAV1 levels in either region (data not shown).

Western blot analysis further verified lower expression of SDPR in B TM compared to W TM (p < 0.0001) with borderline significance of reduced expression of CAV1 in B TM (p = 0.0654, Fig. 3c,d), though no racial difference was detected in CAV1 immunohistochemistry staining. These tissue-specific findings within the TM illustrate that whole genomic data (GWAS)^5^ may lack the granularity needed to fully capture tissue-specific variations. Consequently, these results underscore the importance of focusing on tissue-specific genes and proteins to develop a more nuanced understanding of the racial disparities observed in glaucoma.

Since SDPR protein expression exhibited distinct regional differences between B and W healthy TM, we sought to determine if SDPR RNA expression also displayed regional variations. To achieve precise and accurate data, we utilized advanced laser microdissection techniques (Fig. 3e,f) to isolate two specific TM tissue regions for qPCR analysis. Results of qPCR analysis showed no significant racial differences in SDPR gene expression among TM regions analyzed, consistent with total TM region qPCR data (Fig. 1h).

Due to the limited size of our POAG patient sample collection, it was not possible to measure SDPR protein levels in these patients. However, we successfully detected SDPR protein expression in healthy donor TM tissues, where it was significantly lower in B compared to W donors (Fig. 3c,d). Moreover, the investigation of protein levels indicated that SDPR protein expression in the corneoscleral and uveal TM regions was significantly lower in healthy B donors compared to W donors (Fig. 3b). Such a lack of direct correlation between RNA and protein expression is a common occurrence in cell biology^30^. Various factors, including post-transcriptional regulation, translation efficiency, and protein stability, can contribute to these disparities^31^. Further studies are warranted to elucidate the intricate mechanisms underlying these differences and their implications for glaucoma pathogenesis.

**Figure 4:**
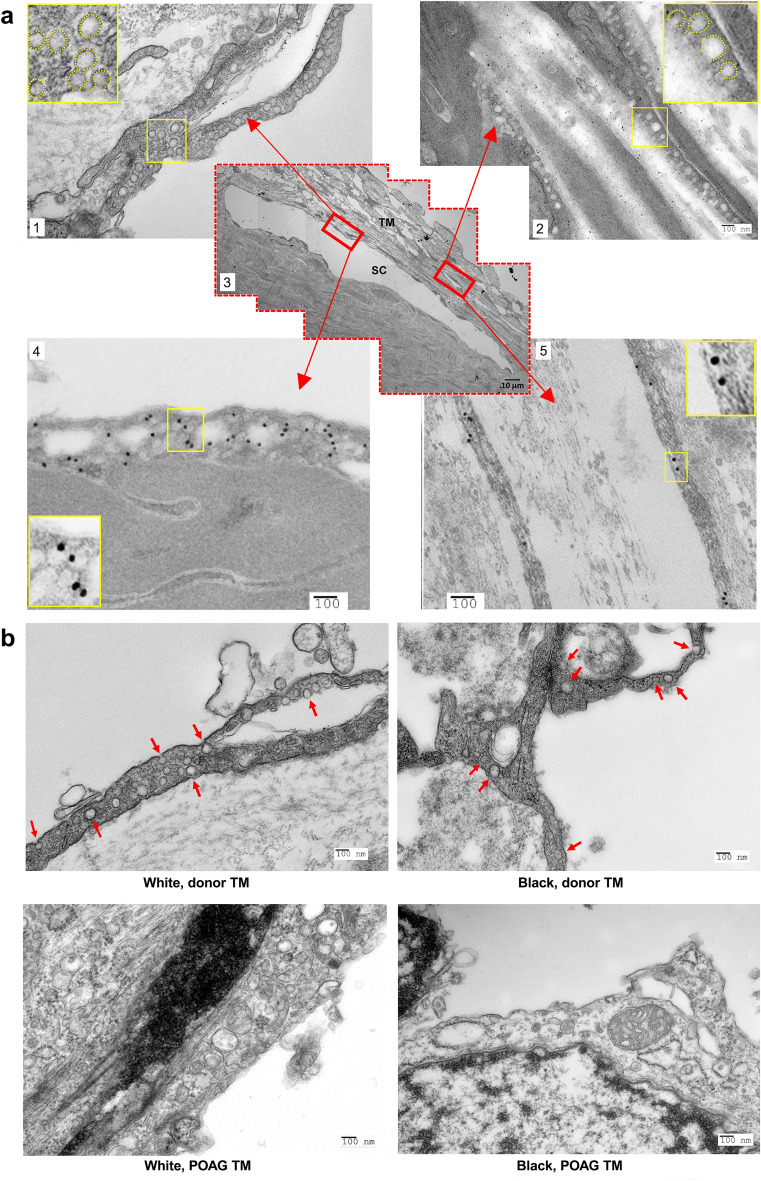
Transmission electron microscopy studies. (**a**) Transmission electron microscopy (TEM) of TM and confirmed SDPR expression on site by immune-gold labeling TEM. a1 and a4-Images of TM adjacent to Schlemm’s canal. a2 and a5-Images at corneoscleral meshwork regions. a1 and a2 (standard TEM) demonstrate singlets and clusters of caveolae (50–100 nm). Abundant invaginated plasmalemmal and caveolar vesicles in cytoplasm are identified (high-magnification of details in yellow windows). a4 and a5: SDPR immuno-gold labeling specifically noted at Schlemm’s canal/JCT region and corneoscleral meshwork, respectively (high-magnification of details in yellow windows). (**b**) Standard TEM image of healthy TM from Black and White donors. (upper panel) Red arrows indicate typical caveolae in cell membrane or cytoplasm. Glaucomatous TM (trabeculectomy specimens without visible caveolae; lower panel). a3-Center assembled image displays entire TM at low magnification. Red box indicates area of observation displayed in other images. Bar: 100 nm. This data represents the fundamental evidence linking SDPR at the molecular level to caveolar ultrastructure morphology in POAG. Additionally, we also detected CAV1 antibody nanoparticles in the caveolae area of healthy TM (Supplementary Materials). These findings shed light on the potential interplay between SDPR expression and caveolae morphology in glaucoma and provide valuable insights into the molecular mechanisms underlying caveolar changes in the disease.

Given the evidence that SDPR expression was reduced in POAG TM, particularly in B patients, this led us to investigate effects on caveolar morphology. To explore the association between SDPR and caveolae morphology, we first employed standard transmission electron microscopy (TEM) to examine TM specimens from healthy donors. Representative TEM images (Fig. 4, legend) of B and W healthy donors revealed abundant caveolae in the TM, characterized by bulb-shaped sub-microscopic organelles (50–100 nm) with double membranes, as visualized with uranyl acetate and Sato's lead staining. Figure 4a-1,a-2 depict a group image representing the caveolar locations, demonstrating normal caveolar structures in both Schlemm canal (SC)/juxtacanalicular (JCT) and TM regions. Figure 4a-4,a-5 demonstrate immuno-gold staining (IGS) TEM, confirming the specific localization of SDPR-binding nanoparticles in the caveolar zones of both SC/JCT and TM regions. Notably, no staining of SC/JCT was observed in the IGS TEM specimens. Furthermore, we compared standard TEM images from glaucomatous TM with healthy TM. We examined three glaucomatous TM (2 W and 1 B) and five healthy TM (3 W and 2 B) samples and found significantly lower caveolae numbers in the glaucomatous TM when compared with healthy TM (Supplementary Fig. 1). Across all regions of the TM block specimens excised from POAG patients by trabeculectomy, we were unable to identify any characteristic morphologic caveolae in POAG TM, which were clearly visualized in healthy TM (Fig. 4b).

Previous studies have meticulously explored the presence of caveolae in SC and the TM, particularly emphasizing the roles of caveolar family members like CAV1/CAV2 in the context of glaucoma and the regulation of aqueous outflow^22,32–35^. These foundational investigations have paved the way for a deeper comprehension of the intricate mechanisms governing eye pressure regulation.

While most existing studies on CAV1/2 are rooted in GWAS,^5^ our research has produced four novel key findings:

1. *SDPR* serves as a tissue-specific gene with elevated expression in both SC and TM. This characteristic positions it as a potential marker for glaucoma research specific to TM.
2. There is a consistent reduction in SDPR expression in primary open-angle glaucoma (POAG) patients when compared to healthy donors, a trend that holds across diverse populations.
3. We observed racial differential expression of SDPR. This variation is evident at RNA levels in POAG patients and at protein levels in the TM of healthy donors.
4. POAG patients exhibit a loss of caveolae ultrastructure compared to healthy donors. This alteration may be linked to the observed reduction in SDPR expression and/or changes in the expression of other genes or proteins within the caveolar family.

These innovative findings not only augment the current body of knowledge but also shed light on previously unexamined relationships between African ancestry and physiological variations in intraocular pressure (IOP) regulation.

Based on these observations, we put forth the hypothesis that diminished SDPR expression in the TM, particularly among patients of African ancestry, may correlate with an increased risk of glaucoma. Such differences in gene expression could directly impact the caveolar structures and functions within the ocular system, thereby affecting TM functionality. Consequently, these changes might lead to elevation of IOP, the most significant risk factor for both the onset and progression of glaucoma. Our research highlights racial disparities in glaucoma susceptibility and sets the stage for additional studies. The aim of future research would be to develop interventions that are specifically designed to further study and address these intricate molecular differences.

This study has several limitations that need to be acknowledged. One major challenge is the absence of an appropriate animal model or cell line to investigate racial disparities in POAG, which requires our reliance on human eye tissue. Human studies are susceptible to biological variability due to factors such as sex, age, medication exposure, prior surgeries, and co-existing conditions, and limited extracted protein quantities—all of which can influence study outcomes^36–38^. POAG surgical specimens may also exhibit anatomical distortions or contamination from adjacent tissues, even with careful preparation, making it challenging to compare them with healthy donor TM in TEM morphology studies. Furthermore, using donor tissue as a "healthy control" introduces uncertainties regarding ocular or systemic diseases and treatments that may impact TM gene expression. Acknowledging the challenges in comparing POAG TM specimens with donor "healthy TM," we must consider that POAG TM specimens are fresh, collected and processed immediately in the operating room, whereas "healthy TM" from donor eyes is preserved in Optisol-GS, potentially leading to postmortem changes that could affect RNA stability and further complicating the interpretation of results.

In this initial study, we did not perform a genomic racial stratification, primarily due to its exploratory nature. We recognize that the lack of detailed racial background information may introduce limitations due to potential racial admixture. We plan to address these concerns in our future research incorporating analyses of genetic variation and mitochondrial haplogroup in order to provide a more comprehensive understanding of the differential gene expression observed between Black and White samples. Despite its limitations, the binary racial classification used in this study, based on self-reporting, is valuable for our studies. However, its reliability and correlation with true ancestral origins are uncertain, potentially impacting genomic assessments^39^.

Nevertheless, this study uncovers new insights into SDPR specific expression in human TM, and the potentially significant role of SDPR in maintaining normal TM function and glaucoma risk, as well as its contribution to racial disparities related to the disease. The findings we have presented here open a new pathway for exploration, suggesting underlying factors that may explain why POAG manifests differently in patients of African ancestry. By delving deeper into these complexities through further research, we aim to pave the way for personalized therapies tailored to molecular differences associated with ancestral background.

## Methods

### Subject enrollment for glaucoma study

This human study was approved by the Institutional Review Board of the Washington University School of Medicine, complying with the Declaration of Helsinki, and Health Insurance Portability and Accountability Act guidelines. Participating patients with POAG undergoing trabeculectomy surgery provided informed written consent after the nature and possible consequences of the study were explained. Additional data from the enrolled patients, encompassing self-reported race, sex, age, glaucoma severity stage, intraocular pressure (IOP) management, medication details, and prior surgical history, were compiled for subsequent analysis.

### Acquisition of POAG patient TM specimens

During a trabeculectomy (glaucoma filtering surgery), a small incision is made in the sclera near the limbus, the border between the cornea and the sclera (Fig. 1C). A partial-thickness scleral flap is then lifted to expose an area of peripheral cornea, trabecular meshwork (TM) and Schlemm’s canal. A section of tissue (approximately 1.5 × 1 mm) is excised and immediately placed in an RNA preservation solution (RNAlater^®^, Thermo Fisher, MA USA). Due to challenges associated with the small size of TM specimens, especially after fixation, shrinkage does not allow performance of immunohistochemistry staining. We acknowledge the limitations of this approach which may include non-TM tissues (sclera, cornea), but these specimens are highly valuable for foundational analyses. Future studies utilizing other surgical techniques such as goniotomy allow more “pure” TM collection. Unlike in vitro studies demonstrating dexamethasone-induced myocilin expression as a specific marker for cultured TM cells^40^, there is no specific TM marker for this TM tissue^41^. For qPCR analysis, detailed information on POAG patient demographics is provided in Supplementary Table 1.

### Acquisition of donor ocular tissues

Trabecular meshwork (TM) tissues from human donors were sourced either directly from Mid-America Transplant (MAT, St Louis, MO), contributing research-qualified corneal tissue, or from Washington University Eye Center, providing residual corneoscleral rims from penetrating keratoplasty operations. All harvested eye tissues were stored in Optisol GS for a period not exceeding 6 days. The collection, handling, and distribution of all donor corneal tissues adhered strictly to the guidelines established by the Eye Bank Association of America (EBAA). Notably, none of the donors had a documented history of ocular disease, and the microdissection process did not reveal any anomalies.

### RNA-seq data acquisition

POAG patients’ trabeculectomy specimens (4 B/4 W) were immediately transferred into RNA stabilization solution (RNAlater™, ThermoFisher Scientific) in the operating suite. One B patient was excluded due to unacceptable RNA quantity. Approximately 2.5 ng of total RNA (MicroRNeasy kit, Qiagen) with RNA integrity number (RIN) score ≥ 8.0 were reverse transcribed and amplified to produce a strand-specific RNA-Seq library (Ovation RNA-Seq System V2; NuGEN Technologies). Ligated fragments were amplified for 12 cycles using primers incorporating unique index tags before sequencing (Illumina Hi-Seq 3000, Illumina) using single reads extending 42 bases. RNA-seq reads were aligned to the Ensembl release 76 assemblies (STAR version 2.0.4b). Gene counts were derived from uniquely aligned unambiguous reads and transcript counts were produced (Sailfish version 0.6.3). Sequencing performance was assessed for the total number of aligned and uniquely aligned reads, genes and transcripts detected, ribosomal fraction known junction saturation and read distribution over known gene models (RSeQC version 2.3). All gene-level and transcript counts were imported into R/Bioconductor package EdgeR and trimmed mean of M-values (TMM) normalization size factors were calculated to adjust for library size differences. The performance of samples was assessed with the Spearman correlation matrix and multi-dimensional scaling plots. Generalized linear models with robust dispersion estimates were created for gene/transcript level differential expression. Differentially expressed genes and transcripts were filtered for FDR adjusted p-values < 0.05. To enhance biological interpretation of the large transcript set, the grouping of genes/transcripts based on functional similarity was achieved using R/Bioconductor packages Genome Assembly Gold-standard Evaluations (GAGE) and Pathview, which were also used to generate pathway maps on known signaling/pathways by Kyoto Encyclopedia of Genes and Genomes (KEGG). Differential expression analysis indicated 437 genes with p < 0.05 and 63 genes with p < 0.01 comparing B and W groups. FDR adjusted p-value adds further statistical stringency to eliminate the probability of false-positive data.

### PCR and qPCR

The glaucomatous RNA samples remaining from RNA-seq analysis then were pooled into two groups, B and W, based on racial self-report. Standard PCR was performed using 3% agarose gel electrophoresis. Approximately 2–5 ng total RNA was extracted using the RNeasy Micro kit (Qiagen Inc., Hilden, Germany). The quality and quantity of total RNA were determined using a Nanodrop spectrophotometer (Thermo Fischer Scientific, Waltham, MA) and 2100 Bioanalyzer system (Agilent, Santa Clara, CA). Total RNA (RNA Integrity Number (RIN) > 8) was reverse transcribed and amplified to produce 3–5 μg cDNA using the WT-Ovation V2 Pico RNA Amplification System (NuGEN Technologies, San Carlos, CA, USA). Amplified cDNA samples were used for the quantitative reverse transcript PCR (qRT-PCR) analyses. The qPCR analysis was performed using KAPA SYBR FAST qPCR Kit for LightCycler480 (Sigma Chemicals, St. Louis, MO, USA) and the LightCycler 480 System (Roche, Indianapolis, IN, USA) according to the manufacturer's protocols. Quantified values for each gene of interest were normalized against the housekeeping gene glyceraldehyde 3-phosphate dehydrogenase (GAPDH) input. Primer pairs are listed in Supplementary Table 2.

### Laser microdissection

The method is the same as our previous study^42^. The corneoscleral rims were embedded in optimal cutting temperature (OCT) compound and stored at − 80 °C. Frozen sections of 10 μm were transferred to glass polyethylene naphthalate (PEN) foil slides (Leica Microsystems, Wetzlar, Germany). The slides were dipped in 70% ethanol for 1 min, washed in RNAase-free water twice for 30 s, rinsed in 95% ethanol, and then stained in Eosin Y.-stained samples were washed in 95% ethanol and dehydrated in 100% ethanol. The TM regions were carefully identified and outlined, then collected by precise laser microdissection to avoid contamination with adjacent tissues (Leica LMD 6000 Laser microdissection system, Figs. 1 and 3).

### Immunohistochemistry staining

Fresh, healthy donor corneoscleral tissues were fixed using 10% Neutral Buffered Formalin for a minimum of three days. The samples were then embedded in paraffin and sectioned at a thickness of 5 μm, with subsequent antigen retrieval. Immunohistochemical staining was performed using SDPR (1:1000, rabbit polyclonal antibody, Thermo Fisher Scientific) and CAV1 (1:500, mouse monoclonal antibody, Thermo Fisher Scientific) following a standard protocol. For cellular nuclear counterstaining, DAPI solution (Thermo Fisher Scientific) was employed before observation using Fluorescence Microscopy at 10×, 20×, and with oil immersion 60× lens. Quantitative fluorescence intensity analysis was conducted using Image J software (Version 1.49u).

### Western Blot

To detect SDPR and CAV1 expression in healthy TM tissues, we collected TM tissues from B and W donors, cut them into smaller fragments, then lysated with 60 μl of RIPA buffer. About 12 μg protein per sample/lane was run with electrophoresis in 4–12% gradient gels and then the proteins were transferred to nitrocellulose membranes for 1 h by Mini Gel Tank (Cat. No. A26977, Thermo Fisher Scientific) and Mini Blot Module (Cat. No. B1000, Thermo Fisher Scientific). Primary antibodies SDPR (rabbit polyclonal antibody, Thermo Fisher Scientific) diluted 1:1000 and CAV1 (mouse monoclonal antibody, Thermo Fisher Scientific) at 1:200 was added overnight at 4 °C. Secondary antibodies IRDye^®^ 800CW Donkey anti-Rabbit IgG and anti-Mouse (LI-COR, Nebraska) were used at 1:20,000. Finally, the Western blot was detected by Odyssey Near-Infrared Western Blots System (LI-COR, Nebraska). The intensity of the bands was normalized to the level of β-actin on the same blots by using a mouse monoclonal antibody (1:10,000; Santa Cruz). Normalization was calibrated by adjusting the intensity of the SDPR and CAV1 bands based on the level of β-actin, then setting the highest normalized intensity for SDPR/CAV1 to 1.0 for each age group. The results with duplicate blots for each experiment were normalized, averaged, and plotted ± SD.

### Western blot analysis of SDPR and CAV1 expression in healthy TM tissues

To evaluate SDPR and CAV1 protein expression of healthy TM tissues, corneal tissues were collected from both B and W donors. Total TM strips were dissected into smaller fragments and lysed using 60 μl of RIPA buffer. Subsequently, approximately 12 μg of protein per sample/lane was separated using electrophoresis on 4–12% gradient gels. The separated proteins were then transferred onto nitrocellulose membranes using the Mini Gel Tank (Cat. No. A26977, Thermo Fisher Scientific) and Mini Blot Module (Cat. No. B1000, Thermo Fisher Scientific).

For immunodetection, primary antibodies SDPR (rabbit polyclonal antibody, Thermo Fisher Scientific) at a dilution of 1:1000 and CAV1 (mouse monoclonal antibody, Thermo Fisher Scientific) at 1:200 was added to the membranes and incubated overnight at 4 °C. Subsequently, secondary antibodies IRDye® 800CW Donkey anti-Rabbit IgG and anti-Mouse (LI-COR, Nebraska) were used at a dilution of 1:20,000. The Western blot signals were visualized using the Odyssey Near-Infrared Western Blots System (LI-COR, Nebraska). To ensure accuracy and consistency, intensity of the bands was normalized to the expression level of β-actin on the same blots, using a mouse monoclonal antibody (1:10,000; Santa Cruz). The averages of blot band intensity were quantified using LI-COR software Image Studio. Normalization was performed by adjusting the intensity of the SDPR and CAV1 bands based on the level of β-actin values, and then setting the highest normalized intensity for SDPR/CAV1 to 1.0 for each group. To obtain reliable results, duplicate blots were used for each sample. The data were normalized, averaged, and presented ± standard deviation (SD).

### Transmission electron microscopy (TEM) and immuno-gold labeling TEM

The TEM study was performed at the Washington University Center for Cellular Imaging utilizing state-of-the-art TEM equipment guided by the center’s expertise and technical support. For TEM, samples were transferred into fixative solution containing 2% paraformaldehyde and 2.5% glutaraldehyde in 0.15 M cacodylate buffer with 2 mM CaCl_2_, pH 7.4 and kept at 4 °C overnight. Samples were then rinsed in cacodylate buffer 3 times and subjected to a secondary fixation for one hour in 1% osmium tetroxide/1.5% potassium ferrocyanide in cacodylate buffer. Samples were then rinsed in ultrapure water 3 times and incubated overnight in an aqueous solution of 1% uranyl acetate at 4 °C. Samples were then washed in ultrapure water 3 times for 10 min in each step, dehydrated in a graded acetone series (50%, 70%, 90%, 100% × 3) for 10 min in each step, and infiltrated with microwave assistance (Pelco BioWave Pro, Redding, CA) into low-viscosity Spurr’s resin. Samples were cured in an oven at 60 °C for 72 h and post-curing, 70 nm thin sections were cut from the resin blocks, post-stained with uranyl acetate and Sato’s lead and imaged on a Transmission Electron Microscope (JEOL JEM-1400 Plus, Tokyo, Japan) operating at 120 kV.

For immunoelectron microscopy, samples were fixed in 4% paraformaldehyde in PBS overnight at 4 °C, washed 3 times in PBS and then incubated in ultrapure water/PBS series (20%, 40%, 60%, 80% and 100%) for 10 min in each step, followed by two 20 min ultrapure water exchanges. Samples were then en bloc stained in 0.5% uranyl acetate for 15 min in the dark followed by three rinses, 10 min each, in ultrapure water. Samples were partially dehydrated to 85% ethanol in the following series: 20% and 40% ethanol for 10 min each, 50% and 70% ethanol for 15 min each and finally 85% ethanol for 10 min. Subsequently, samples were infiltrated into LR White resin (2 parts LR White resin mixed with 1 part 85% ethanol for 1 h, 100% LR White resin for 1 h followed by 100% LR White resin overnight). The next morning, samples were placed in a fresh aliquot of LR White resin and rotated for 1 h prior to polymerization in a 55 °C oven overnight. For immunogold labeling, ultrathin sections (50–80 nm) were cut and collected on nickel grids. Grids were floated on modified HEPES buffer (100 mM NaCl, 30 mM HEPES, 2 mM CaCl2 pH 7.0) for 10 min followed by incubation in aldehyde quenching solution (1 × modified HEPES solution, 50 mM dl-lysine monochloride, 50 mM glycine, 50 mM ammonium chloride) for 30 min. Grids were then incubated in blocking buffer (1% bovine albumin in modified HEPES buffer) for 30 min followed by incubation in primary antibody (SDPR polyclonal, #PA5-34521, Invitrogen) in blocking buffer (1:100 dilution) for 30 min. Grids were then washed 10 times with blocking buffer for 2 min each and incubated in secondary antibody (8 nm polyclonal, #711-205-152, Jackson Immuno Research) in blocking buffer (1:15 dilution) for 30 min. Following this, grids were rinsed 10 times in blocking buffer, 2 min each, then rinsed with 3 quick passes through drops of ultrapure water, post stained with 2% uranyl acetate for 25 min, and imaged on a Transmission Electron Microscope (JEOL JEM-1400 Plus, Tokyo, Japan) operating at 120 kV.

### Statistical analysis and reproducibility

All healthy donor TM experiments were performed in triplicate and the mean values were utilized for analysis. Due to limited samples from POAG patients, repeated experiments were not possible. Identified outliers were removed from the data analysis. Statistical parameters including the exact n for each experiment and statistical significance are reported in figure legends. Unpaired Student’s t-test by using GraphPad Prism 9 (GraphPad Software, La Jolla, CA) was performed. p < 0.05 will be considered statistically significant. For RNAseq data, all gene-level and transcript counts were analyzed by R/Bioconductor package EdgeR and R/Bioconductor package Limma. Differentially expressed genes and transcripts were filtered for FDR adjusted p-values < 0.05 and the results were achieved using R/Bioconductor packages Genome Assembly Gold-standard Evaluations (GAGE) and Pathview, which were also used to generate pathway maps by signaling/ pathways by Kyoto Encyclopedia of Genes and Genomes (KEGG). The datasets used and/or analyzed during the current study are available from the corresponding author upon reasonable request and will be de-identified prior to release.

Software:

- GraphPad Prism 9 (GraphPad Software, La Jolla, CA).
- R/Bioconductor package EdgeR.
- R/Bioconductor package Limma.

### Supplementary Information

Supplementary Figure 1.

Supplementary Information.

Supplementary Table 1.

Supplementary Table 2.

## Data Availability

The datasets generated during and/or analyzed during the current study are available from the corresponding author on reasonable request.
