# Supplementary material for: SDPR expression in human trabecular meshwork and its potential role in racial disparities of glaucoma
Source: Sci Rep. 2024 May 4;14:10258. doi: 10.1038/s41598-024-61071-w (PMC11069504; doi:10.1038/s41598-024-61071-w)
Supplement: Supplementary file 1 — Supplementary Figure 1. [file 41598_2024_61071_MOESM1_ESM.pdf]

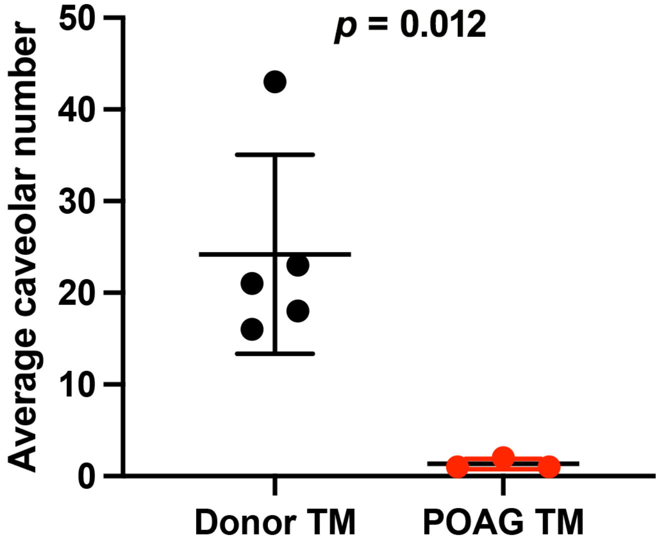

Supplementary Figure 1. Caveolae quantity from transmission electron microscopy (TEM) images. Each dot represents one subject with mean number of caveolae numbers calculated from >10 TEM images. Mean ± SD. TM= trabecular meshwork, POAG= Primary open angle glaucoma
