# Supplementary material for: SDPR expression in human trabecular meshwork and its potential role in racial disparities of glaucoma
Source: Sci Rep. 2024 May 4;14:10258. doi: 10.1038/s41598-024-61071-w (PMC11069504; doi:10.1038/s41598-024-61071-w)
Supplement: Supplementary file 2 — Supplementary Information. [file 41598_2024_61071_MOESM2_ESM.pdf]

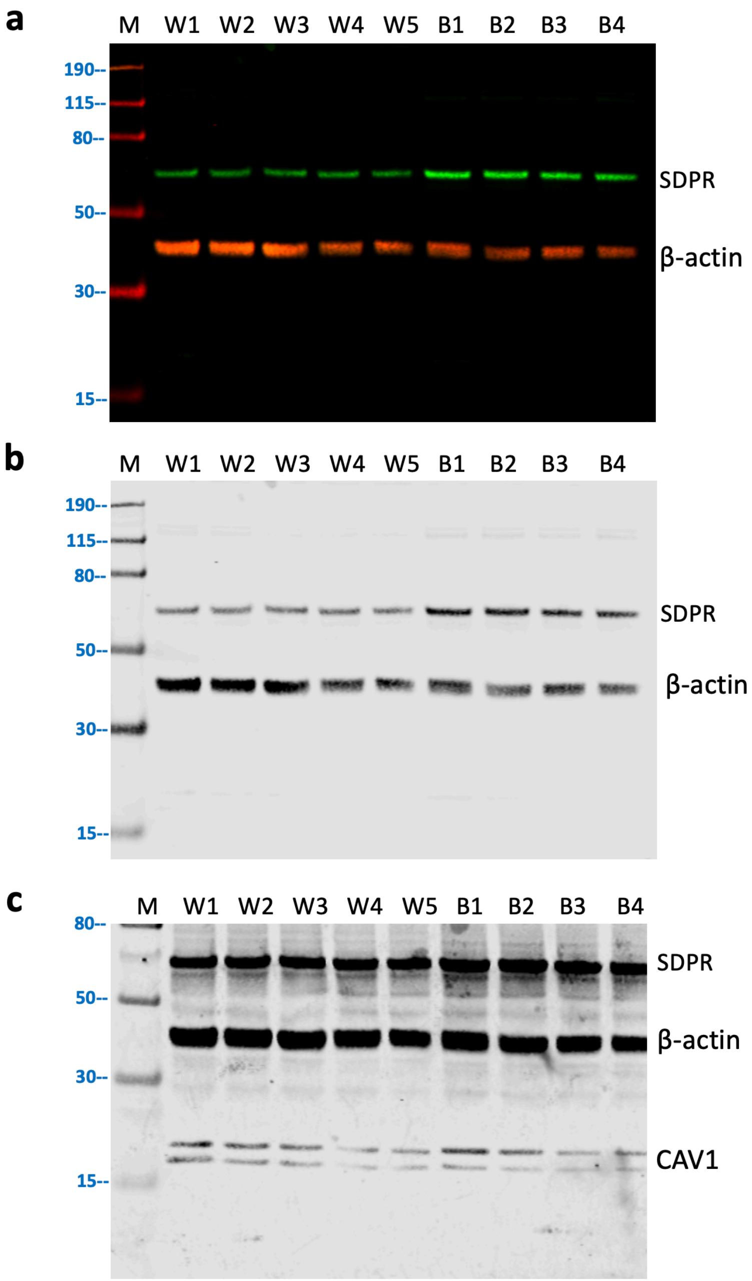


**Western blot of SDPR/CAV1**- a) Original scan image with blot exposure time = 30 seconds. Green bands indicate SDPR (68-70 kDa); Orange bands indicate beta-actin (42 kDa). b) Conversion to white/black. c) Over-exposure up to 10 minutes, double bands (21-24kDa) indicate CAV1 expression. Note: Final quantitative analysis based on one blot with two exposure times (Figure 3).
