# Supplementary material for: SDPR expression in human trabecular meshwork and its potential role in racial disparities of glaucoma
Source: Sci Rep. 2024 May 4;14:10258. doi: 10.1038/s41598-024-61071-w (PMC11069504; doi:10.1038/s41598-024-61071-w)
Supplement: Supplementary file 3 — Supplementary Table 1. [file 41598_2024_61071_MOESM3_ESM.pdf]

Supplementary Table 1. Patient Demographics

	Black group (n=7)	White group (n=12)	*p*
**Age (years)**	67.1 (95% CI: 57.2~77.1)	71.8 (95% CI: 67.4~76.2)	0.2598
**Sex**			
Male	3	8	
Female	4	4	
**Lens Status**			
Phakic	4	7	
Pseudophakic	3	5	
**Mean pre-operative glaucoma medications**	2.3 (95% CI: 1.6~3.0)	2.4 (95% CI: 1.8~2.8)	0.8981
**Glaucoma severity ***	2.9 (95% CI: 2.5~3.2)	2.7 (95% CI: 2.4~3.0)	0.3913
**Previous surgery**			
Cataract extraction with lens implant	3	5	
Selective laser trabeculoplasty	1	1	

CI- confidence interval

* Glaucoma severity- Hodapp-Anderson-Parrish classification[27]
