# Supplementary material for: SDPR expression in human trabecular meshwork and its potential role in racial disparities of glaucoma
Source: Sci Rep. 2024 May 4;14:10258. doi: 10.1038/s41598-024-61071-w (PMC11069504; doi:10.1038/s41598-024-61071-w)
Supplement: Supplementary file 4 — Supplementary Table 2. [file 41598_2024_61071_MOESM4_ESM.pdf]

Supplementary Table 2. List of primer sequences of caveolae-associated genes.

Gene	Forward (5′-3′)	Reverse (5′-3′)
**CAV1 (Caveolin 1)**	gcacttgcaaccgtctgtta	ctcctcccccatcttctttc
**CAV2 (Caveolin 2)**	gcctaatggttctgccttca	cgtcctacgctcgtacacaa
**Cavin 1 (PTRF)**	aagaagctggaggtcaacga	tttgctgatgctcagtttgg
**Cavin 2 (SDPR)**	ttgatggcataacctattcagc	acacagccattgttggttc
**Cavin 3 (SRBC)**	cttgtgccttgtcccaaaat	cacaggactgggcttaagga
**GAPDH**	cagccgagccacatcgctcagacaccat	ctttaccagagttaaaagcagccctgg
